# Mitochondrial DNA markers reveal high genetic diversity and strong genetic differentiation in populations of *Dendrolimus kikuchii* Matsumura (Lepidoptera: Lasiocampidae)

**DOI:** 10.1371/journal.pone.0179706

**Published:** 2017-06-29

**Authors:** Qiulei Men, Guoxi Xue, Dan Mu, Qingling Hu, Minyi Huang

**Affiliations:** 1 School of Life Sciences, Provincial Key Laboratory of the Biodiversity Study and Ecology Conservation in Southwest Anhui, Research Center of Aquatic Organism Conservation and Water Ecosystem Restoration in Anhui Province, Anqing Normal University, Anqing, Anhui, P. R. China; 2 School of Food and Bioengineering, Zhengzhou University of Light Industry, Zhengzhou, Henan, P. R. China; 3 School of Chemistry and Environment, Weinan Normal University, Weinan, Shaanxi, P. R. China; National Cheng Kung University, TAIWAN

## Abstract

*Dendrolimus kikuchii* Matsumura, 1927 is a serious forest pest causing great damage to coniferous trees in China. Despite its economic importance, the population genetics of this pest are poorly known. We used three mitochondrial genes (COI, COII and Cytb) to investigate the genetic diversity and genetic differentiation of 15 populations collected from the main distribution regions of *D*. *kikuchii* in China. Populations show high haplotype and nucleotide diversity. Haplotype network and phylogenetic analysis divides the populations into three major clades, the central and southeastern China (CC+SEC) clade, the eastern China (EC) clade, and the southwestern China (SWC) clade. Populations collected from adjacent localities share the same clade, which is consistent with the strong relationship of isolation by distance (r = 0.74824, P = 0.00001). AMOVA analysis indicated that the major portion of this molecular genetic variation is found among the three groups of CC+SEC, EC and SWC (61.26%). Of 105 pairwise FST comparisons, 93 show high genetic differentiation. Populations of Puer (PE), Yangshuo (YS) and Leishan (LS) are separated from other populations by a larger genetic distance. Distributions of pairwise differences obtained with single and combined gene data from the overall populations are multimodal, suggesting these populations had no prior population expansion in southern China. The nonsignificant neutral test on the basis of Tajima’ D and Fu’s Fs, and the lack of a star-shaped haplotype network together with the multiple haplotypes support this hypothesis. Pleistocene climatic fluctuations, combined with the host specificity to *Pinus* species, made these regions of south China into a refuge for *D*. *kikuchii*. The high level of population genetic structuring is related to their weak flight capacity, their variations of life history and the geographic distance among populations.

## Introduction

Simao pine moth, *Dendrolimus kikuchii* Matsumura, 1927, is a destructive forest pest with an extensive range across southern China. Larvae attack various coniferous trees and regular outbreaks occur. These outbreaks are mainly on *Pinus langhianensis* Chev, *P*. *yunnanensis* Franch, *P*. *massoniana* Lambert, *P*. *armandi* Franch and *Keteleeria evelyniana* Mast. [1,2]. *D*. *kikuchii* larvae consume, on average, 7486.6 mm of *P*. *langhianensis* pine needles to complete their development [3]. During outbreaks, a large amount of pine needles are consumed, giving the appearance of forest fire damage. Larval damage from a large infestation of *D*. *kikuchii* can reduce the yield of timber, resin and cones, and affect the growth rate of pines, even resulting in tree death [1,2]. The Simao pine moth shows differences in ecological preferences among areas in China. One generation of *D*. *kikuchii* occurs in areas with short periods of optimal environment, such as in the middle region of Yunnan and Guizhou provinces. In Zhejiang, Fujian, and the southwest region of Yunnan provinces, two generations occur. In different counties of Yunnan province, such as Jingdong and Anning, the life histories of *D*. *kikuchii* also show significant variation [1].

Genetic diversity and genetic structure in insects can be affected by many factors, such as host plant species, chemical controls, geographic distance and geographic barriers [4–11]. For some lepidopterous species, the genetic diversity and genetic structure are related to their migration capacity and number of generations [12–15]. Despite the economic and landscape threats of the *D*. *kikuchii* to pine trees in the southern parts of China and the need to establish control strategies for this pest, it has been unclear whether analogous associations between these factors and genotype patterns may be present among *D*. *kikuchii* populations.

Pleistocene climatic fluctuations are thought to have a great effect on this species’ distribution and historical demography [16]. South China was considered as a key area of some refugia for many relict rare species during the Pleistocene glaciation [17,18]. Zhang et al [19] studied the geohistory of *Dendrolimus punctatus*, a sympatric species of *D*. *kikuchii*, based on these geological events. They found that *D*. *punctatus* settled down in south China with the spread of masson pine during the Pleistocene. However, to our knowledge, there is no published report on the population history of *D*. *kikuchii*, and also no studies on the population history of *Dendrolimus* species based on molecular data.

In this study, we use three mitochondrial genes to (i) investigate the genetic diversity and genetic differentiation of 15 populations collected from the main distribution regions of *D*. *kikuchii* in southern China, and infer the demographic history of this pest, and (ii) to test the hypothesis that geographical isolation and biological characters, such as life history, are significant factors underlying genetic variation in Chinese *D*. *kikuchii*.

## Material and methods

### Ethics statement

There is no endangered or protected species involved in this study. No specific permissions were required for the described field studies for this widespread forest pest. We confirm that the locations are not privately owned or otherwise protected.

### Sampling

**Fig 1:**
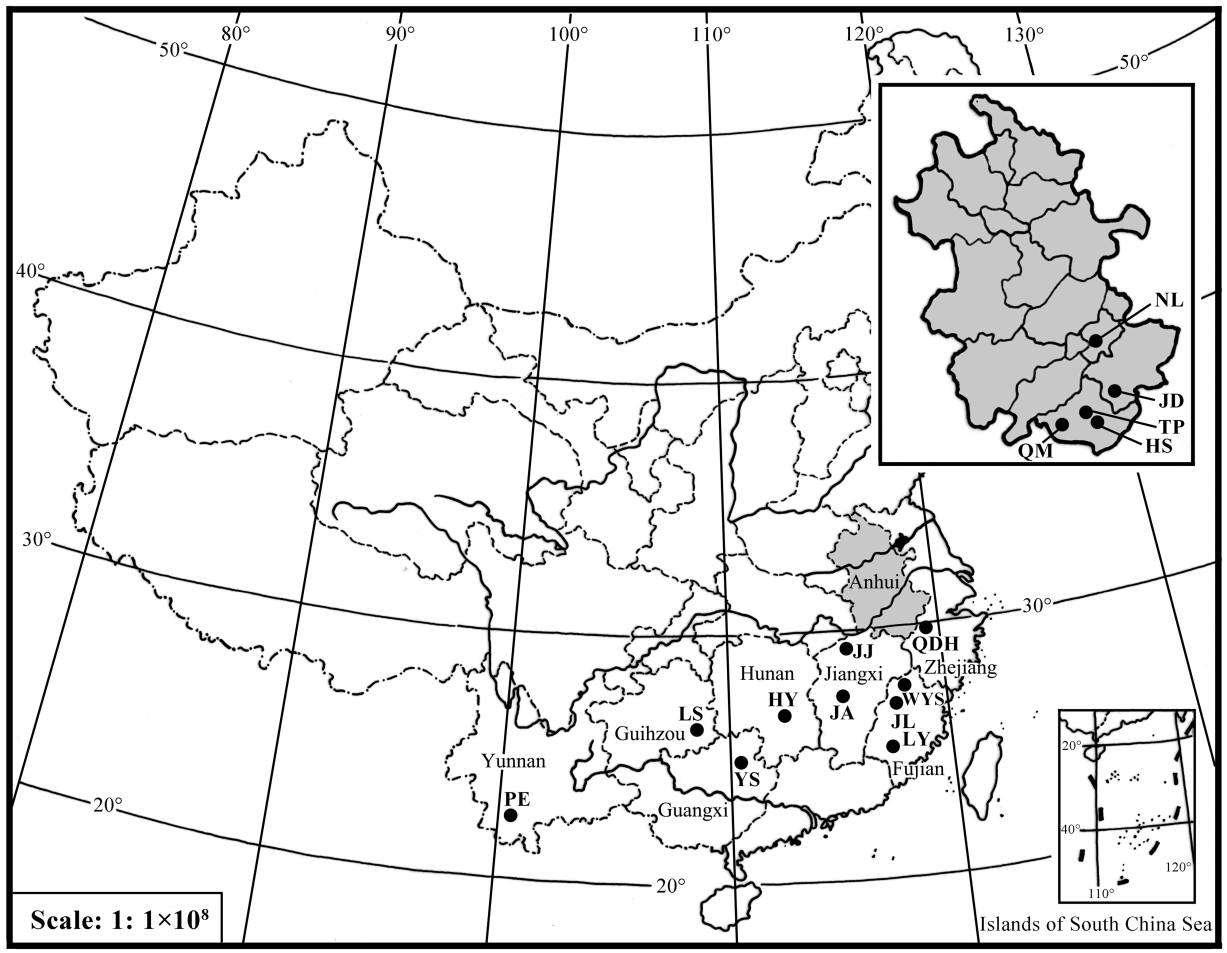
Sample locations for the 15 *D*. *kikuchii* populations from China, the codes for *D*. *kikuchii* populations are explained in Table 1; the gray region represents the geographic distribution of five *D*. *kikuchii* populations in Anhui.

A total of 182 individuals were collected from 15 locations during 2013 to 2016 within the *D*. *kikuchii* China distribution range (Table 1 and Fig 1). Different instar larvae and adult moths were sampled. For larvae, only one individual was collected per tree. For adult moths, pheromone traps were used in the pine forest (>1 ha) and only one moth from each trap was sampled in order to avoid sampling errors. All sampled insects were immersed in absolute ethyl alcohol, and then stored at -20°C prior to genetic analysis.

**Table 1:** Sampling information of *D*. *kikuchii* in China.

Province	Location	Population code	Latitude	Longitude	Sample size	Sample date
Fujian	Longyan	LY	25°04′N	116°59′E	8	2016.06.19
	Jiangle	JL	26°42′N	117°27′E	4	2014.04.16
	Wuyishan	WYS	27°45′N	117°54′E	15	2016.05.01
Hunan	Hengyang	HY	26°56′N	112°43′E	32	2014.06.13
Jiangxi	Jian	JA	27°00′N	114°49′E	19	2016.05.20
	Jiujiang	JJ	30°22′N	118°25′E	4	2016.04.06
Zhejiang	Qiandaohu	QDH	29°35′N	119°00′E	10	2014.08.07
Guangxi	Yangshuo	YS	24°44′N	110°27′E	4	2014.08.19
Yunnan	Puer	PE	22°50′N	100°46′E	32	2013.10.25
Guizhou	Leishan	LS	25°04′N	108°11′E	4	2015.06.01
Anhui	Nanling	NL	30°55′N	118°15′E	6	2013.09.20
	Huangshan	HS	30°05′N	118°12′E	26	2014.06.07
	Qimen	QM	29°53′N	117°41′E	5	2014.04.01
	Taiping	TP	30°19′N	118°00′E	7	2014.04.15
	Jingde	JD	30°22′N	118°25′E	6	2014.05.15

### DNA extraction and amplification

Genomic DNA was extracted from the last proleg of the larvae or from any leg of the adult using the DNeasy Tissue Kit (QIAGEN, Hilden, Germany). Extraction was performed according to the bench protocol for animal tissues. Mitochondrial COI (LCO1490, HCO2198, [20]), COII (Eva, Patrick, [21]), and Cytb (generated for codling moth, unnamed [22]) were selected for use in this study. Polymerase chain reactions (PCR) were performed using a S1000 Thermal Cycler (BIO-RAD, Hercules, CA, USA) in a total volume of 20 μl, containing 10 μl 2×PCR Super Master Mix (Biotool, Shanghai, China), 0.25 μM of each primer, and 1 μl genomic DNA (10–30 ng/μl). PCR amplification was employed with denaturation at 95°C for 10 min, followed by 40 amplification cycles consisting of 95°C for 30 s, primer-specific annealing temperature of 53°C (COI), 52°C (COII and Cytb) for 1 min, 72°C for 45 s, and then a final step at 72°C for 10 min. Amplified products were purified and sequenced by Tianyi Huiyuan Biotechnology Co., Ltd.

## Data analysis

The sequences were preliminarily aligned using the CLUSTAL X program [23]. Sequences of COI (646), COII (675), Cytb (700) of *D*. *kikuchii* were deposited in the NCBI GenBank (GenBank accession numbers: COI, MF155667-MF155697; COII, MF155698-MF155737; Cytb, MF155738-MF155760) (data in S1–S3 Text). Multiple sequences of COI, COII and Cytb were concatenated to yield a total length of 2021 bp. The haplotype network of *D*. *kikuchii* was analyzed using a median-joining algorithm in the program Network 4.6 [24]. A neighbour-joining (NJ) tree was built using NJ tree subroutine in NEIGHBOUR within PHYLIP 3.5 [25], and the parameters were expanded 1,000 times. The CONSENSE subroutine within PHYLIP was then applied to generate a consensus NJ tree that provided bootstrap support at each node. The tree was visualized using treeview version 1.6.6 software. DnaSP 5.0 [26] were performed to calculate number of polymorphic sites (S), number of haplotypes (H), haplotype diversity (Hd), nucleotide diversity (Pi), Tajima’s D (D), and Fu’s Fs (Fs). Analysis of molecular variance (AMOVA) was performed using the ARLEQUIN version 3.5 based on the combinations of the three gene sequences [27], along with calculating pair fixation indices (FST). The pairwise genetic distances were calculated by MEGA 6.0 [28] based on the Kimura-2-parameter model [29]. Referring to the criterion for genetic differentiation by Wright (1978) [30], we defined genetic differentiation as low for FST<0.05, moderate for 0.05<FST<0.15, high for 0.15<FST<0.25, and very high for FST>0.25 [31]. In order to test isolation by distance (IBD), the matrices of genetic distance FST/(1-FST) and the geographic distance (ln) between all 15 sampling populations were compared using the Mantel test with 10,000 permutations [32]. This analysis was performed using the ZT software package [33]. For examining demographic history, the distribution of pairwise differences between individual sequences was analyzed by means of mismatch distribution analysis using DnaSP 5.0 [26]. The formula, Tau = 2 ut was used to detect the time of population size changes [34]. The nucleotide substitution rate in mitochondrial DNA was 2.3% per million years (MY) as suggested in Knowles et al. (2000) [35].

## Results

### Genetic diversity

**Fig 2:**
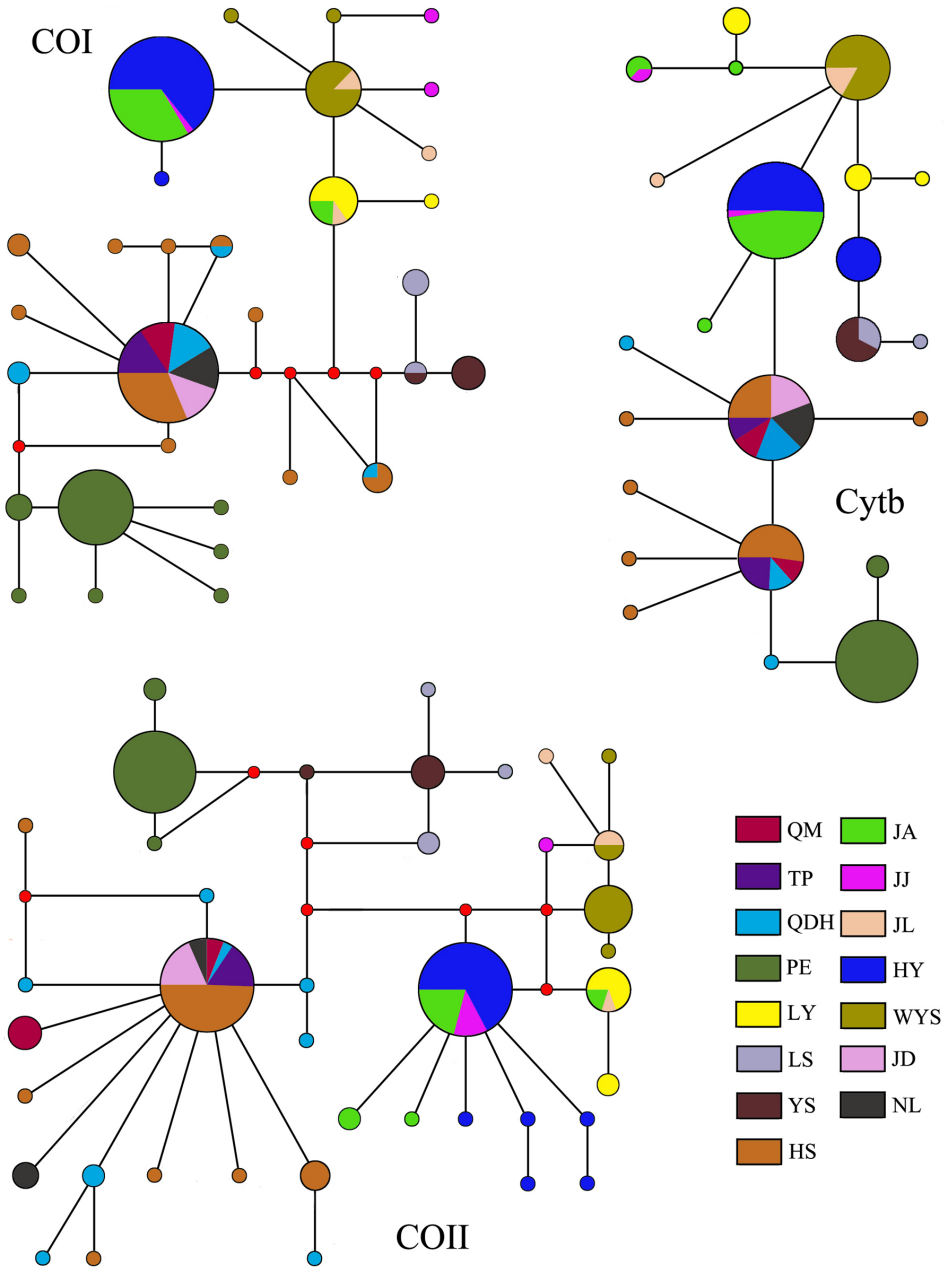
Median-Joining network based on the single genes of COI, COII and Cytb haplotypes. Each circle represents a haplotype, and the area of a circle is proportional to the number of observed individuals. Colors within the nodes refer to the *D*. *kikuchii* sampling regions.

For concatenated sequences, the haplotype diversity ranged from 0 to 1 with a average of 0.940, while the nucleotide diversity ranged from 0 to 0.00251 with a average of 0.01451 (Table 2). All populations displayed large numbers of mitochondrial haplotypes, with a total 31 haplotypes obtained for COI, 40 haplotypes for COII, and 23 haplotypes for Cytb. Seven, six and four common haplotypes were shared for COI, Cytb and COII respectively (Fig 2). The median-joining network demonstrated a high genetic diversity for populations of *D*. *kikuchii* in China. Of 73 examined haplotypes, 68 were unique. Most populations lacked a common haplotype.

**Table 2:** Parameters of genetic diversity based on mitochondrial sequence data of 15 populations of *D*. *kikuchii*.

Population (Abbreviation)	Combined gene			
	S	Hd	Pi	H
Longyan (LY)	6	0.929	0.00127	6
Jiangle (JL)	9	1.000	0.00223	4
Wuyishan (WYS)	6	0.648	0.00050	6
Hengyang (HY)	11	0.613	0.00057	8
Jian (JA)	10	0.604	0.00113	6
Jiujiang (JJ)	10	0.833	0.00251	3
Qiandaohu (QDH)	20	1.000	0.00244	10
Yangshuo (YS)	2	0.333	0.00033	2
Puer (PE)	9	0.442	0.00042	8
Leishan (LS)	5	1.000	0.00130	4
Nanling (NL)	1	0.600	0.00030	2
Huangshan (HS)	35	0.942	0.00176	18
Qimen (QM)	2	0.600	0.00059	2
Taiping (TP)	3	0.714	0.00057	2
Jingde (JD)	0	0	0	1
Total	150	0.940	0.01451	73

This table includes population code, number of polymorphic sites (S), number of haplotypes (H), haplotype diversity (Hd), nucleotide diversity (Pi)

### Genetic differentiation

**Fig 3:**
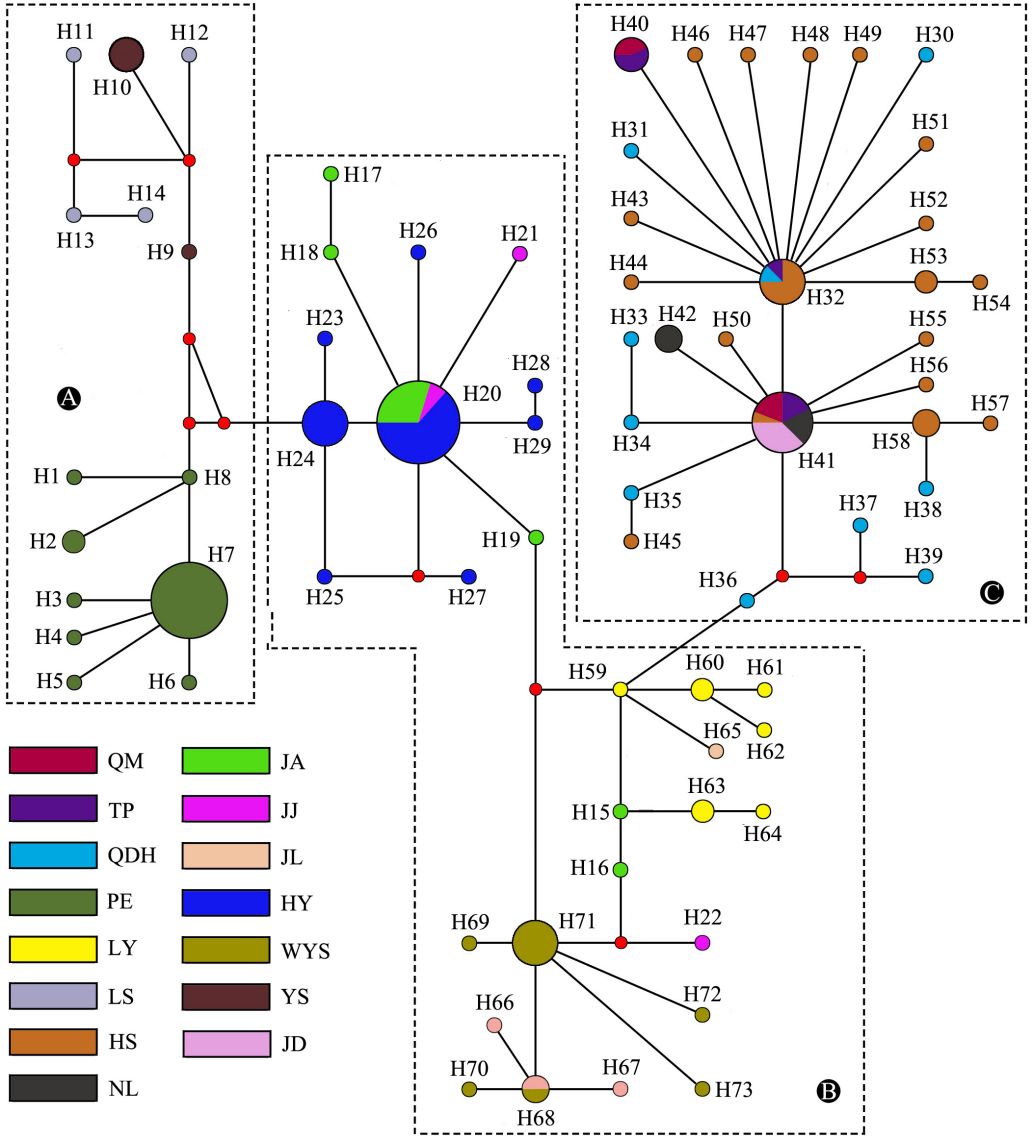
Median-Joining network based on the combined gene of COI, COII and Cytb haplotypes. Each circle represents a haplotype, and the area of a circle is proportional to the number of observed individuals. Colors within the nodes refer to the *D*. *kikuchii* sampling regions. A, B, and C indicate the three clades obtained.

The Median-Joining network of the haplotypes can be divided into three major clades (Clades CC+SEC, EC and SWC) (Fig 3). All haplotypes in Clade CC+SEC were obtained from samples from central China and southeastern China including Hunan, Jiangxi, and Fujian provinces, while all haplotypes in Clade EC were from eastern China including Anhui and Zhejiang provinces. The haplotypes in Clade SWC included samples from southwestern China and covered Yunnan, Guizhou and Guangxi provinces. Overall, populations collected from adjacent localities or the same province shared the same clade. However, the Median-Joining network of the haplotypes based on single gene of COI and Cytb was not in line with the result from the combined genes. This may due to less nucleotide variation in both single genes.

**Fig 4:**
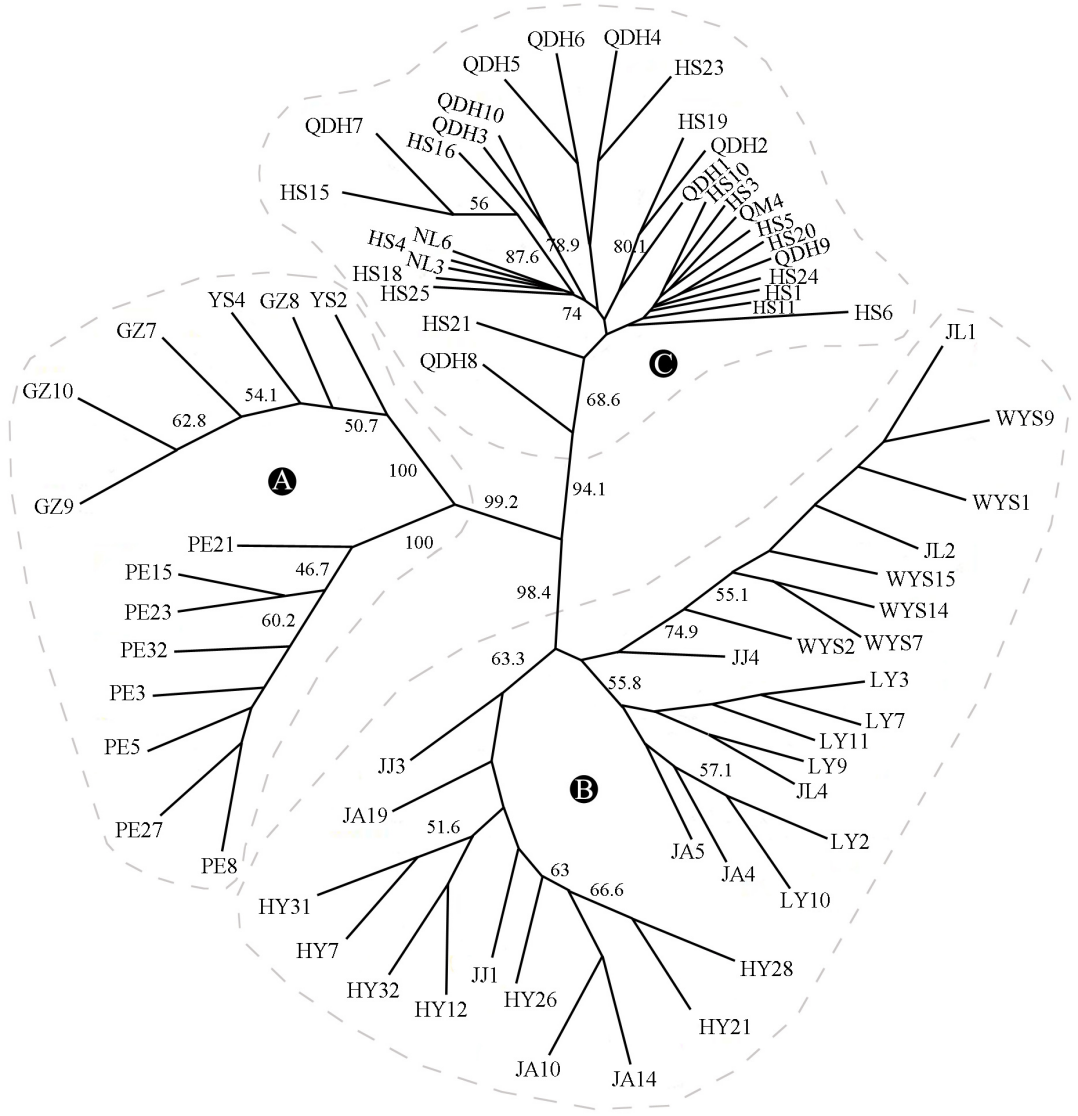
Unrooted NJ phylogenetic tree of *D*. *kikuchii* based on the combination of the three gene sequences, estimated with PHYLIP using Kimura-2-parameter (1000 replications). The bootstraps were shown near the nodes. A, southwestern China clade; B, central and southeastern China clade; C, eastern China clade.

Phylogenetic reconstruction using the Kimura-2-parameter resulted in a consensus NJ tree with comparatively higher bootstrap values. The populations were divided into three major clusters, CC+SEC, EC and SWC, which comply well with the results from the haplotype network (Fig 4).

The values of pairwise FST range from 0 to 0.98921. Of 105 comparisons, 93 showed high genetic differentiation. The pairwise FST values between JA and JJ populations were less than 0.15, indicating moderate genetic differentiation. The pairwise FST values between the populations from Anhui and Zhejiang provinces, ranged from 0 to 0.06363, indicating low genetic differentiation. FST values among populations from Anhui Province range from 0 to 0.11845, suggesting a relatively low genetic differentiation (Table 3). This is consistent with the results of the clustering analysis based on a Median-Joining network.

**Table 3:** Pairwise FST (below diagonal) and genetic distance (above diagonal) based on mitochondrial sequence data of 15 populations of *D*. *kikuchii*.

Populations	YS	LS	PE	HY	JA	JJ	LY	QDH	QM	TP	JD	JL	NL	HS	WYS
YS		0.001	0.032	0.015	0.016	0.016	0.016	0.017	0.016	0.016	0.016	0.017	0.017	0.017	0.016
LS	**0.50183**		0.032	0.016	0.016	0.016	0.017	0.017	0.016	0.016	0.016	0.017	0.017	0.017	0.016
PE	**0.98699**	**0.98409**		0.035	0.036	0.036	0.036	0.034	0.035	0.035	0.034	0.037	0.034	0.035	0.036
HY	**0.96470**	**0.95783**	**0.98555**		0.001	0.002	0.004	0.009	0.009	0.009	0.008	0.004	0.009	0.009	0.003
JA	**0.94212**	**0.92592**	**0.98172**	**0.15596**		0.002	0.003	0.009	0.009	0.009	0.008	0.003	0.008	0.009	0.003
JJ	**0.92542**	**0.87855**	**0.98241**	**0.31462**	0.04251		0.004	0.009	0.009	0.009	0.008	0.004	0.009	0.009	0.003
LY	**0.94535**	**0.92293**	**0.98352**	**0.78962**	**0.62791**	**0.51950**		0.009	0.009	0.009	0.008	0.003	0.009	0.009	0.003
QDH	**0.89997**	**0.87190**	**0.97362**	**0.88554**	**0.81229**	**0.73154**	**0.78903**		0.002	0.002	0.001	0.009	0.002	0.002	0.009
QM	**0.97232**	**0.94363**	**0.98695**	**0.93328**	**0.88359**	**0.83314**	**0.88277**	0.00000		0.000	0.000	0.009	0.001	0.001	0.008
TP	**0.97135**	**0.94872**	**0.98688**	**0.93451**	**0.89058**	**0.85657**	**0.89253**	0.05049	0.00000		0.000	0.009	0.001	0.001	0.008
JD	**0.98980**	**0.96903**	**0.98921**	**0.94126**	**0.90191**	**0.88147**	**0.91128**	0.00153	**0.29412**	**0.39893**		0.008	0.000	0.001	0.008
JL	**0.93646**	**0.89343**	**0.98354**	**0.77249**	**0.57759**	**0.32164**	**0.49575**	**0.74463**	**0.84983**	**0.86993**	**0.89709**		0.009	0.009	0.001
NL	**0.98090**	**0.95813**	**0.98803**	**0.93755**	**0.89446**	**0.86311**	**0.89925**	0.06363	**0.32174**	**0.40954**	**0.40000**	**0.87828**		0.001	0.008
HS	**0.90978**	**0.89650**	**0.96992**	**0.87922**	**0.83112**	**0.79529**	**0.82309**	0.03713	0.00000	0.00127	0.05501	**0.80177**	0.11845		0.009
WYS	**0.97151**	**0.95951**	**0.98734**	**0.82091**	**0.71737**	**0.63749**	**0.74795**	**0.85392**	**0.93544**	**0.93644**	**0.95268**	**0.25305**	**0.94402**	**0.85192**	

The values in bold represent P<0.05.

The values of pairwise genetic distance between PE population and other populations range from 0.032 to 0.037, indicating high genetic differentiation.

Similar high values were observed in comparisons of the LS population with other populations, which range from 0.016 to 0.032. Comparisons of the YS population with other populations range from 0.015 to 0.032. The values of genetic distance among the remaining populations are less than 0.01 (Table 3).

AMOVA results indicate that the major portion of the molecular genetic variation is found among groups (61.26%). Exact tests showed a significant genetic variance on all three levels (P<0.001) (Table 4).

**Table 4:** Analysis of molecular variance of populations.

Source of variation	d. f.	Sum of squares	Variance components	Percentage of variation	P value
Among groups	2	1672.749	12.13831 Va	61.26	P<0.001
Among populations within groups	12	782.032	6.72706 Vb	33.95	P<0.001
Within populations	164	155.756	0.94973 Vc	4.79	P<0.001
Total	178	2610.536	19.81510		

The Mantel test for the 15 populations revealed a positive correlation between genetic distances and geographic distances (r = 0.74824, P = 0.00001), suggesting that isolation by distance had a limiting effect on gene flow.

### Mismatch distribution

**Fig 5:**
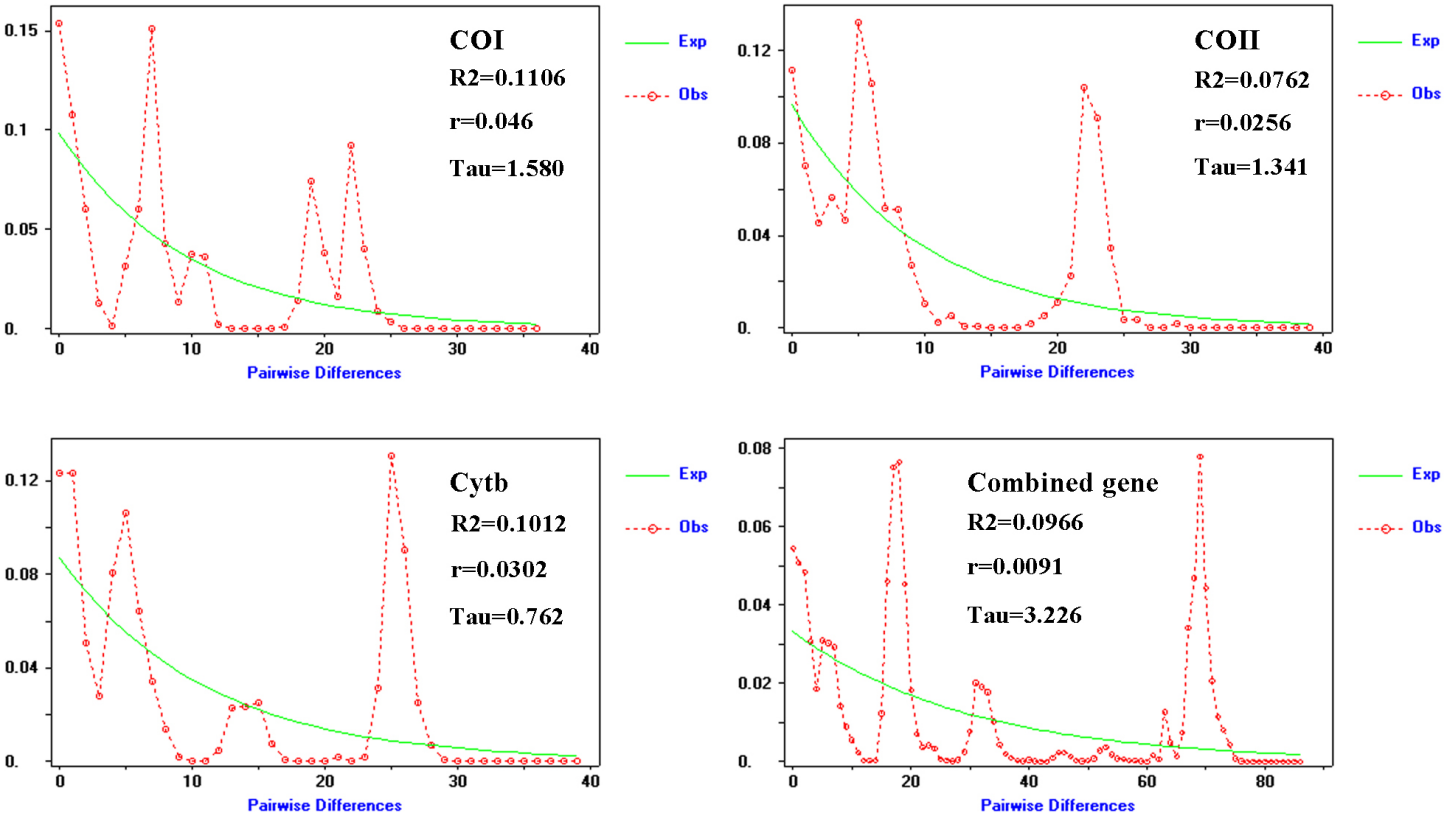
Observed and expected mismatch distributions for *D*. *kikuchii* in China, based on COI, COII, Cytb, and combined gene sequences.

The results of the combined gene analysis show that Tajima’ s D values are significantly positive with a value of 0.24250, but are not significant in most specific populations (P<0.05 in HS, PE and HY, P>0.05 in the rest of the populations). Fu’s F statistic was significantly negative with a value of -5.132 (P>0.1) (Table 5). At the population level, HS, HY and PE populations have negative and significant Tajima’ s D and Fu’s Fs values, whereas HS, HY and PE populations show a bimodal distributions, suggesting that expansion events were not detected with this analysis (data not shown). Distributions of pairwise differences (mismatch distributions) obtained with the single and combined gene data from the overall populations were multimodal, suggesting that the populations of *D*. *kikuchii* in southern China did not experience population expansion (Fig 5). A nonsignificant neutral test based on Tajima’ D and Fu’s Fs support this interpretation. The time of reaching a stable population size are estimated to be 26500 (COI), 21600 (COII), and 11800 (Cytb) years ago.

**Table 5:** Parameters of the neutral test based on mitochondrial sequence data of 15 populations of *D*. *kikuchii*.

Population (Abbreviation)	Combined gene			
	Tajima’s D		Fu’s Fs	
	*D*	*P*	*Fs*	*P*
Longyan (LY)	0.5185	>0.10	-1.980	>0.05
Jiangle (JL)	-0.8294	>0.10	-0.664	>0.10
Wuyishan (WYS)	-1.5856	>0.05	-2.782	<0.05
Hengyang (HY)	-1.8270	**<0.05**	-3.202	**<0.05**
Jian (JA)	-1.0770	>0.10	-0.630	>0.10
Jiujiang (JJ)	-0.5281	>0.10	1.557	>0.10
Qiandaohu (QDH)	-1.4796	>0.10	-6.049	**<0.05**
Yangshuo (YS)	-1.1320	>0.10	0.952	>0.10
Puer (PE)	-1.9080	**<0.05**	-4.601	**<0.05**
Leishan (LS)	-0.2125	>0.10	-1.414	>0.10
Nanling (NL)	1.4451	>0.10	0.795	>0.10
Huangshan (HS)	-2.2918	**<0.01**	-11.469	**<0.05**
Qimen (QM)	1.4588	>0.10	1.688	>0.10
Taiping (TP)	1.6500	>0.10	0.263	>0.10
Jingde (JD)	0	-	0	-
Total	0.2425	>0.10	-5.132	>0.10

This table includes population codes, Tajima’s D (D), and Fu’s Fs (Fs). Bold type values indicate statistical significance (P<0.05).

## Discussion

Using three mitochondrial genes, we investigated the genetic diversity and structure of 182 individuals of 15 *D*. *kikuchii* populations sampled throughout their main areas of distribution in China. The results show a high genetic diversity and high level of genetic structuring of *D*. *kikuchii* in the sampled areas.

All populations displayed large numbers of mitochondrial haplotypes, with a total 31 haplotypes obtained for COI, 40 haplotypes for COII, and 23 haplotypes for Cytb, of which seven, six and four were common haplotypes shared respectively. Of 73 examined haplotypes based on combined genes, 68 were unique and did not share the same ancestral haplotype. High numbers of private haplotypes and lack of ancestral haplotype suggest that *D*. *kikuchii* could be a species native to China. In a previous study, Zheng et al. [36] indicated that these two features can be considered as indicators for native species, especially for native species with a low dispersal capacity, such as *Grapholita molesta* Busck (Lepidoptera: Tortricidae) and *Chilo suppressalis* (Walker) (Lepidoptera: Pyralidae). Although the origin of *D*. *kikuchii* remains uncertain, the higher genetic diversity we observed in the present study supports our assumption that China is part of the original range of *D*. *kikuchii*.

Based on the results of three mitochondrial genes, including AMOVA analysis, haplotype network and phylogenetic analysis, we conclude that *D*. *kikuchii* populations were geographically structured in three regions: eastern China, southwestern China, and central China along with southeastern China. The sampling localities of southwestern China are isolated by the Wumeng, Leigong, and Fanjingshan Mountains, while sampling localities of central China are isolated by the Hengshan and Dabieshan Mountains, which have acted as substantial barriers to gene flow. Populations from central China and southeastern China shared common haplotypes; this is in accordance with the results of phylogenetic analysis. Although there is a geographical barrier formed by Wuyishan Mountain between these two regions, a transportation network increases the gene flow among *D*. *kikuchii* populations. In Lepidoptera species, dispersal patterns also influence genetic variation [37,38]. *D*. *kikuchii* is generally regarded as a sedentary species based upon previous studies of its flight capacity, with a sphere of activity extending over 10~20 km [1, 3]. The weak flight capacity of *D*. *kikuchii* can reduce gene flow among populations. The IBD relationship (r = 0.74824, P = 0.00001) in the present study supports this hypothesis. Similar trends have been identified for many sedentary species, such as *Chilo suppressalis* (Walker) [15] and *Carposina sasakii* Matsumura [18]. The same result was reported by Weng et al. [39]. They found that the genetic variation of *D*. *punctatus* populations that ranged over five adjacent regions in Zhejiang province were low using the ISSR-PCR marker.

Analysis of genetic distance indicate that populations of Puer (PE), Yangshuo (YS) and Leishan (LS) are separated from other populations. In fact, *D*. *kikuchii* caterpillars in Puer and Yangshuo populations turn into adults one moth earlier than those of other populations. And in Guizhou, only one generation occurs, while two generations occur in the rest of the provinces [1]. These variations in life history may contribute to the significant genetic differentiation. Moreover, the areas of distribution of *D*. *kikuchii* in China occur across very complex topography (tall mountains, plain and basin), different climates (temperate and tropical climates), different agricultural landscapes and forest types. A high level of population differentiation presents a high potential for adaptation to different environmental conditions [40] and high reproductive rates [41], which allow the moth to form locally differentiated populations. Similar results have been found for *Chilo suppressalis* [15] and *Reticulitermes chinensis* [42].

Distributions of pairwise differences (mismatch distributions) obtained with COI, COII, Cytb, and combined gene data from the overall populations are multimodal, suggesting that the populations of *D*. *kikuchii* did not experience population expansion. The lack of a star shape for the haplotype network together with the existence of multiple haplotypes support such a hypothesis. This also explains why there are so many private haplotypes in different geographical populations. The times when population histories stabilized are estimated to be 26500 (COI), 21600 (COII), and 11800 (Cytb) year ago. This range is within the late Pleistocene, which was characterized by climatic oscillations between warm and cold periods [43]. Over 42 thousand years ago, *Pinus massoniana*, the favorite host plant for *D*. *kikuchii*, gradually spread into the warmer south China, a condition they would prefer [19]. These events impacted the distribution of *D*. *kikuchii*, with these regions of south China gradually becoming the refuge for *D*. *kikuchii*. During the late Pleistocene, some regions of south China were not covered by large ice sheets [44]. In addition, south China comprises a mountainous mosaic area and has the potential to host microclimatic zones that are probably capable of providing some key refuges for many relict species [45]. Considering the lack of an ancestral haplotype and strong isolation-by-distance relationships of this species, we can conclude that *D*. *kikuchii* in south China has arisen in separate refuges and experienced parallel evolutions. Similar results have been found for grasshoppers [46], and the blue manakin [47].

Future population genetic research on *D*. *kikuchii* in China should cover a larger area and larger number of sampled individuals, as well as use nuclear genes as markers to provide a greater understanding of genetic structure. Additional research needs to be done to detail the geographic origins of *D*. *kikuchii* in China and its spread through China.

## Conclusion

Using three mitochondrial genes, we investigated the genetic diversity and structure of 182 individuals of *D*. *kikuchii* sampled throughout its main distribution areas in China. The results show high genetic diversity and a high level of genetic structuring of *D*. *kikuchii* in sampled areas. The high level of population genetic structuring is related to the weak flight capacity of the *D*. *kikuchii*, variations in its life history and the geographic distance among populations. Distributions of pairwise differences (mismatch distributions) obtained with COI, COII, Cytb, and combined gene data indicate the populations of *D*. *kikuchii* in southern China did not experience population expansion. These genetic data not only provide us with an understanding of population genetics for such a secondary species, but also provide guidance for pest management strategies.

## Supporting information

S1 Text Submission data of COI gene segment (numbered 2018976) in NCBI GenBank. (TXT)

S2 Text Submission data of COII gene segment (numbered 2018977) in NCBI GenBank. (TXT)

S3 Text Submission data of Cytb gene segment (numbered 2018978) in NCBI GenBank. (TXT)
